# Research on Sika Deer Behavior Recognition Based on YOLOv11 Lightweight SDB-YOLO Model for Small Sample Learning

**DOI:** 10.3390/ani16010108

**Published:** 2025-12-30

**Authors:** He Gong, Zuoqi Wang, Jinghuan Hu, Yan Li, Longyan Liu, Yanhong Yu, Juanjuan Fan, Ye Mu

**Affiliations:** 1 College of Information Technology, Jilin Agricultural University, Changchun 130118, China; 2 Jilin Province Agricultural Internet of Things Technology Collaborative Innovation Center, Changchun 130118, China; 3 College of Electronic Science and Engineering, Jilin University, Changchun 130012, China

**Keywords:** sika deer behavior, pose recognition, YOLOv11, lightweight model

## Abstract

In the breeding scene, limited by the small number of samples and environmental interference such as illumination occlusion, sika deer behavior recognition still faces challenges such as insufficient feature representation and weak cross-scale modeling ability. To this end, this study builds a lightweight improved model SDB-YOLO based on YOLOv11n. Firstly, the FPSC module is proposed to enhance the correlation between multi-scale features through the shared convolution mechanism, so as to significantly improve the quality of feature fusion under the condition of small samples. Secondly, the Ghost feature generation and dynamic convolution strategy are introduced into the C3k2 module to construct the C3_GDConv structure, so as to strengthen the fine-grained behavior pattern modeling ability and reduce redundant calculations. In addition, the CBAM attention mechanism is added to the neck of the network to further improve the ability of key information extraction and enhance the discrimination of feature expression. Finally, the EfficientHead was used to replace the original detection head to obtain a more robust training process and higher detection accuracy in small-sample scenarios. Experimental results show that SDB-YOLO achieves 90.2% detection accuracy with only 4.3 GFLOPs of calculation, which achieves significant performance improvement compared with YOLOv11n, and verifies the effectiveness and lightweight advantages of the proposed method in small-sample special animal behavior recognition tasks.

## Simple Summary

In practical breeding scenarios, automatic behavior recognition for sika deer often lacks accuracy due to limited behavioral samples collected, coupled with factors like lighting variations and occlusions. This study proposes a more compact new model, SDB-YOLO, to address this issue. The model effectively utilizes limited data to capture more representative behavioral information from images while reducing computational overhead. Simultaneously, the newly designed recognition architecture enhances training stability and facilitates higher accuracy in small-sample environments. Experimental results demonstrate that SDB-YOLO achieves a 90.2% recognition accuracy while maintaining extremely low computational requirements, outperforming existing baseline models.

## 1. Introduction

Animal behavior recognition systems have been widely applied in ecological research, encompassing various techniques such as normal spectral imaging, hyperspectral imaging, ultraviolet, and inf rared spectroscopy. The application of vegetation indices not only serves agricultural practices but also underpins fundamental research describing plant community biodiversity. However, these methods primarily target static objects, whereas animal studies have accumulated extensive telemetry data for tracking the movements of farm and commercial animals. In recent years, researchers have begun developing video surveillance systems and behavioral recognition methods for large animals. These tools assess individual signaling, social structures, stress responses, learning and training capabilities, and aid in selecting and evaluating breeding traits. Even subtle changes in facial expressions and behaviors can reveal an animal’s state and intent [1,2,3,4], demonstrating the practical value and scientific significance of automated behavior recognition in herd management.

As an important economic animal, sika deer products such as antlers, blood, and meat are widely used in traditional Chinese medicine and related industries. Their health status and behavioral patterns directly impact production efficiency. During breeding management, behavioral recognition assists breeders in more accurately monitoring and managing animal behavior, thereby enhancing management standards [5]. Current animal behavior recognition technologies primarily fall into two categories: contact-based and non-contact-based. Contact-based methods deploy sensors on animal body parts such as the lower jaw or legs to collect data, with sensor placement significantly affecting recognition accuracy [6,7,8,9]. Non-contact methods rely on computer vision and deep learning models to analyze and recognize animal behaviors [8,10]. This study aims to achieve precise identification of sika deer behaviors using non-contact methods to enable intelligent farming management. The most advanced work in this field is summarized in Table 1. Yujin Gong et al. [11] addressed the structural bottlenecks of YOLOv5s by proposing a Dynamic Re-parameterization Enhancement DRE module to replace the traditional C3 module. This module integrates deformable convolutions with RepConv architecture, while adopting a stage-customized EfficientViT-m0 as the improved backbone network. Additionally, they designed a difficulty-aware detection head. Mengjie Zhang et al. [12] introduced FasterNet based on YOLOv8n to reduce the complexity of the model, and combined Hybrid Local Channel Attention MLCA module, SIoU loss function, and CARAFE reconfiguration strategy to reduce the number of parameters while maintaining high accuracy. Yizhi Luo et al. [13] replaced the original backbone network of YOLOv8 with GhostNet and integrated the FasterNet Block with Enhanced Multi-scale Attention EMA into the C2f module, simultaneously constructing the lightweight multi-path detection ELMD. This approach demonstrated significant advantages in both efficiency and accuracy. Chen Chen et al. [14] introduced the Sobel-edge Feature Aggregation module (SEFA) and the CSP-dualconv Feature Refinement module (DCFR) in YOLOv11. These enhancements strengthened edge and fine-grained feature representation at the backbone network level, improving the mAP metric. Bai et al. [15] constructed the Res2 backbone to integrate multi-scale receptive fields, enhancing YOLOv3’s backbone for multi-scale features in cattle behavior. The optimized YOLOv3 detector combines global positional information within images, while the Global Context Predict detection head is designed to improve performance in recognizing cattle behavior within crowded environments. Previous studies have made significant progress in the identification and behavioral detection of common farmed animals such as poultry. However, research in the field of specialty economic animals remains relatively scarce. The previous behavior recognition methods for special economic animals only focus on the accuracy, but do not pay attention to the training speed and the number of parameters of the network. They usually increase the training speed at the cost of increasing the number of parameters [16]. This has obvious limitations in applications on resource-constrained devices. In order to better meet the intelligent monitoring needs of special economic animals, this study proposes a lightweight and efficient sika deer behavior recognition model based on YOLOv11 model, which improves the detection accuracy, reduces the model complexity, and enhances its deployability in actual breeding scenarios.

The primary contributions of this paper can be summarized as follows:

(1) The FPSC (Feature Pyramid Shared Convolution) module is proposed to solve the problem of insufficient information transmission of traditional FPN/PAFPN in small-sample scenarios by introducing a shared convolution structure between multi-scale features to strengthen cross-level feature correlation. This significantly enhances the stability of feature fusion.
(2) Based on C3k2, a C3_GDConv structure combining dynamic convolution and Ghost feature generation is constructed. Through adaptive convolution weight adjustment and low-redundancy feature generation mechanism, the model’s sensitivity to fine-grained behavior differences is improved, and the computational overhead is reduced while the feature expression efficiency is enhanced.
(3) The CBAM attention mechanism is introduced at the neck of the network to enhance the feature response ability of key regions through the joint modeling of channel and spatial dimension, so that the model can obtain more discriminative feature expression under complex breeding conditions such as illumination change and occlusion.
(4) The EfficientHead is used to replace the original detection head structure, which makes the parameter organization of the detection branch more reasonable, and shows better gradient transfer and convergence stability under small-sample training conditions, so as to improve the overall accuracy and robustness of target action recognition.
(5) For the sika deer behavior recognition task with only more than one thousand samples, the overall lightweight and structure refinement are realized from the backbone, neck to the detection head. The final constructed SDB-YOLO achieves 90.2% detection accuracy with only 4.3 GFLOPs of calculation. The effectiveness and lightweight advantages of the method in small-sample special animal behavior recognition are verified.

## 2. Materials and Methods

### 2.1. Materials

#### 2.1.1. Data Sources

The behavioral data on sika deer used in this study was obtained from fixed surveillance footage at the Shuangyang Deer Farm in Jilin Province. The farm houses approximately 200 sika deer, with each enclosure containing 8 to 10 deer. The data collection system comprised multiple Xiaomi surveillance cameras (Shanghai Chuangmi New Energy Technology Co., Ltd., Shanghai, China). Videos were uniformly stored in MP4 format. Each camera featured dual 4-megapixel resolution and supported up to 2.5 K ultra-high-definition quality, enabling clear recording of the deer’s postural changes and behavioral details. Cameras were mounted at fixed positions around the breeding area, primarily covering deer pens, activity enclosures, and feeding zones. The collection process involved no contact with or disturbance to the animals. Data was gathered from May to October, fully documenting the deer’s coat color transition from summer’s bright spots to winter’s cryptic markings. This ensures the sample represents key seasonal characteristics throughout the year and enhances the reliability of behavioral recognition results. This timeframe was chosen to encompass critical phases of both coat color and behavioral shifts, ensuring data reflects seasonal variations while minimizing interference from fur changes on recognition accuracy. Color analysis can serve as an additional indicator for assessing animal condition [17], though implementing this analysis requires additional resources.

**Figure 1:**
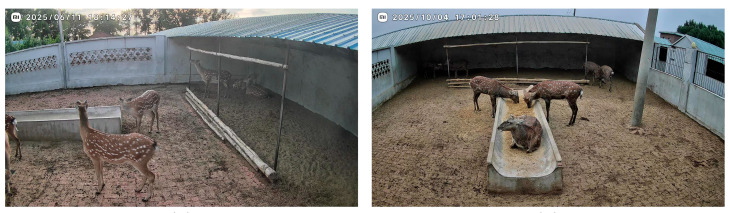
Behavior image of sika deer in the surveillance video of deer house.

During the initial collection phase, we manually screened the raw surveillance footage, discarding segments where the target was absent, severely obstructed, or of poor image quality. Ultimately, 1000 valid video clips were retained. These encompass a variety of typical behaviors exhibited by sika deer in their daily farming environment, including standing still, walking, feeding, and lying down. The dataset exhibits a certain degree of imbalance in the distribution of different behaviors, a characteristic consistent with the objective patterns of animal behavior frequency in real breeding scenarios. Figure 1 shows some examples of video frames in the deer house monitoring environment, including the scenes of Mei feeding at the feed trough, standing and lying on her stomach, reflecting the real breeding environment under different regions, posture changes, and lighting conditions.

In order to construct a sika deer behavior image dataset suitable for model training, the filtered videos were further preprocessed. Firstly, low-quality frames caused by blur, out-of-focus, and abnormal illumination were removed. Then, the frame extraction strategy is used to extract representative key image frames, and the redundant images with highly similar content are removed to improve the effectiveness and diversity of the dataset. In view of the fact that the data came from a single deer farm and the scene changes were relatively limited, this study introduced the idea of small-sample learning in the data construction stage to alleviate the impact of limited sample size on the generalization ability of the model. Finally, 570 high-quality effective images were obtained, and the data augmentation operation was used to expand to 1200 image samples for model training and validation.

#### 2.1.2. Dataset Construction

**Figure 2:**
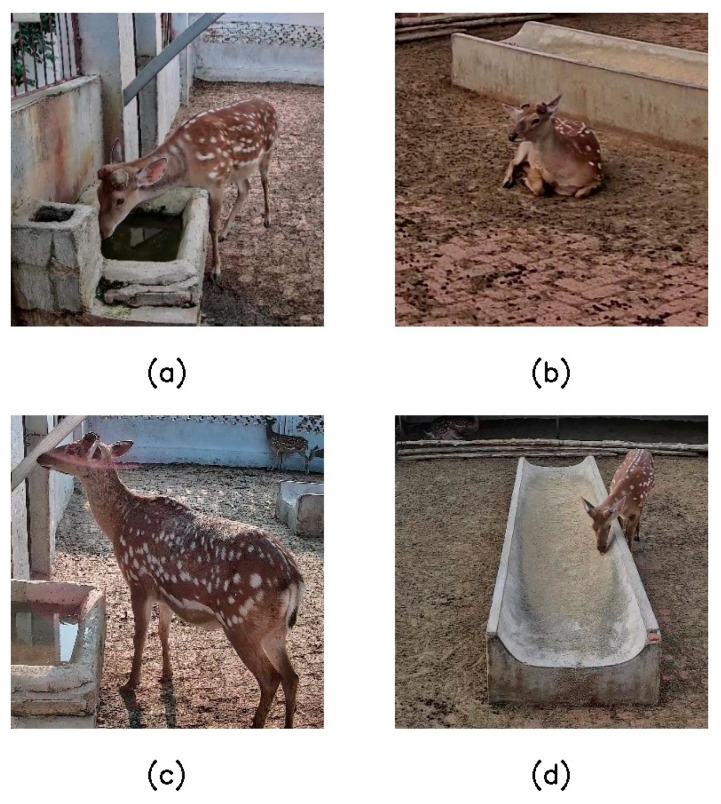
Typical sample images of different classes of behaviors: (**a**) sika deer in drinking water; (**b**) sika deer lying prone; (**c**) sika deer standing; (**d**) sika deer that are eating.

Combined with the behavioral characteristics of sika deer in the actual breeding environment, this paper selected four representative daily behaviors of standing, lying, eating, and drinking as the research objects, and the specific definitions and discrimination criteria of each behavior are shown in Table 2. Figure 2 shows typical sample images of different types of behaviors, showing the manifestations of each behavior in different poses and perspectives. In the process of data collection, due to the differences in the occurrence frequency of different behaviors in the natural state of sika deer, the original data showed unbalanced characteristics in the category distribution. Among them, the frequency of drinking behavior in the breeding scenario is low, and the number of relevant samples is relatively limited, which poses certain challenges for model training.

**Figure 3:**
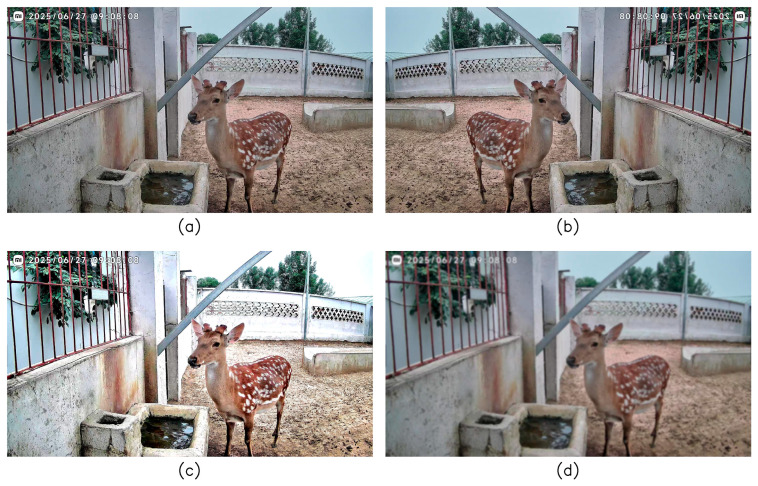
Dataset augmentation diagram: (**a**) original image; (**b**) side-to-side flipping; (**c**) enhanced temporal illumination variation; (**d**) ambiguous movements.

In order to mitigate the impact of class imbalance and improve the adaptability of the model in practical application scenarios, this paper introduces a variety of data augmentation strategies to the dataset during the training phase. Firstly, the basic enhancement methods such as horizontal flip, vertical flip, and color perturbation were used to enhance the adaptability of the model to pose changes and appearance differences. Secondly, aiming at the problems of dynamic interference, target scale change and unstable lighting conditions common in outdoor breeding environment, motion blur, multi-scale fusion enhancement and temporal illumination change enhancement are further introduced. An example of relevant data augmentation is shown in Figure 3. After data augmentation processing, the dataset was divided into training set, validation set, and test set according to a fixed proportion, with 1140, 347, and 110 samples, respectively. The number of labels of each category in the specific dataset is shown in Table 2. For the low-sample category, this paper combines the small-sample learning method in the subsequent model training to reduce the influence of the difference in the number of samples on the recognition performance. Through the above process, build a suitable for sika deer behavior recognition task of dataset, providing the data for model training and performance evaluation.

### 2.2. Methods

#### 2.2.1. Sika Deer Behavior-YOLO (SDB-YOLO)

**Figure 4:**
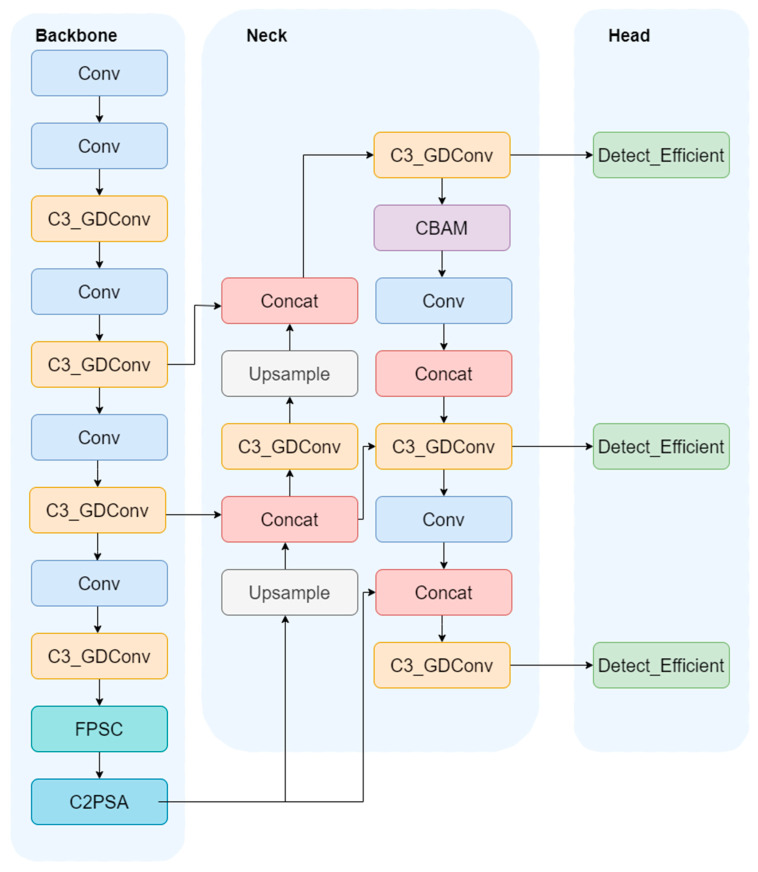
SDB-YOLO Model.

This study’s improvements synergistically enhance performance across multiple levels—from feature extraction and fusion to the detection head mechanism—enabling the model to maintain robust behavioral recognition capabilities even in small-sample scenarios. This provides a reliable solution for intelligent monitoring in real-world production environments. The improved model is illustrated in Figure 4:

The SDB-YOLO model in this study consists of three parts: backbone, neck, and head. For the sika deer behavior recognition task with small samples, backbone gradually extracts stable multi-scale features through multi-layer convolution and C3_GDConv module, and adds FSPC and C2PSA at the end to enhance the perception ability of subtle action differences. The neck part uses upsampling, downsampling, and feature concatenation to effectively fuse the features of different levels. At the same time, CBAM is added at key positions, so that the model can still highlight the key information related to behavior in the case of limited samples. After further integration by C3_GDConv, the fused features are, respectively, input into the Detect_Efficient module in head for the recognition of multi-scale behavioral targets. The overall structure is lightweight and improves the recognition reliability in small-sample scenarios.

#### 2.2.2. FPSC Module

**Figure 5:**
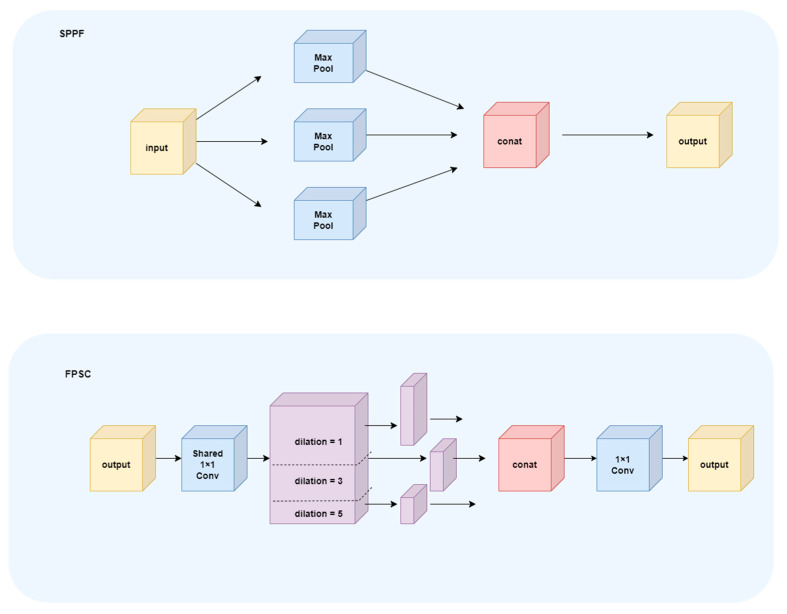
SPPF and FPSC modules.

The SPPF module in YOLOV11 model expands the receptive field by three Max pooling and feature splicing. Its structure is relatively fixed and its feature expression ability is limited, so it is difficult to mine multi-scale information in small-sample scenes. In order to better extract the behavior information of sika deer, this study creatively designed a lightweight and efficient multi-scale feature fusion module FPSC (Feature Pyramid Shared Convolution), which has stronger spatial resolution retention ability and learnable multi-scale spatial convolution feature fusion. Compared with SPPF module, the feature expression is stronger and the spatial information is more abundant. The SPPF module and the improved FPSC module are shown in Figure 5.

First, we adopted the approach of shared convolution kernels and parallel processing with multiple dilation rates [18]. Input features were compressed through channel reduction using 1 × 1 convolutions, aiming to reduce computational overhead in subsequent convolutions. Subsequently, a shared 3 × 3 convolution kernel is used to perform continuous convolution under different dilations (dilations = 1,3,5), so that the receptive field of the feature is gradually expanded.

(1) $$\begin{array}{c} F_{d}=W_{*d}X^{\prime },\;d\in \left\{1,3,5\right\} \end{array}$$

*d denotes the dilated convolution with dilation rate d;

Shared convolution means: W_(d = 1) = W_(d = 3) = W_(d = 5) = W

This reduces the number of parameters significantly:

(2) $$\begin{array}{c} {Params}_{FPSC}={Params}_{3\times 3} \end{array}$$

The traditional multi-scale convolution needs to set the convolution kernel for each scale independently, while the FPSC module uses the same convolution kernel for all scales, which effectively avoids redundant calculations and greatly reduces the amount of parameters and computational cost. After multi-scale feature generation, the features obtained by convolution with different expansion rates are concatenated along the channel dimension to form rich spatial semantic combinations. Then, feature fusion is performed by 1 × 1 convolution. For the feature fusion strategy, a method based on multi-scale feature fusion and dynamic feature integration [19] is adopted. The formula of dynamic weighting mechanism is as follows.

(3) $$\begin{array}{c} F_{out}=\sum_{d\in \left\{1,3,5\right\}}\beta_{d}F_{d} \end{array}$$

(4) $$\begin{array}{c} \beta_{d}=\frac{\exp\left(g_{d}\right)}{\sum_{k\in \left\{1,3,5\right\}}\exp\left(g_{k}\right)} \end{array}$$

Enables the model to dynamically adjust the fusion weight of features across different scales based on input content, thereby avoiding expression limitations caused by static structures. By introducing the shared convolution kernel, multi-expansion rate receptive field expansion and dynamic fusion mechanism, FPSC can generate multi-scale feature expressions containing rich local structural information and global semantic relationships under the premise of lightweight. Compared with SPPF, FPSC is more stable and efficient in small-sample action recognition tasks.

#### 2.2.3. C3_GDConv Module

**Figure 6:**
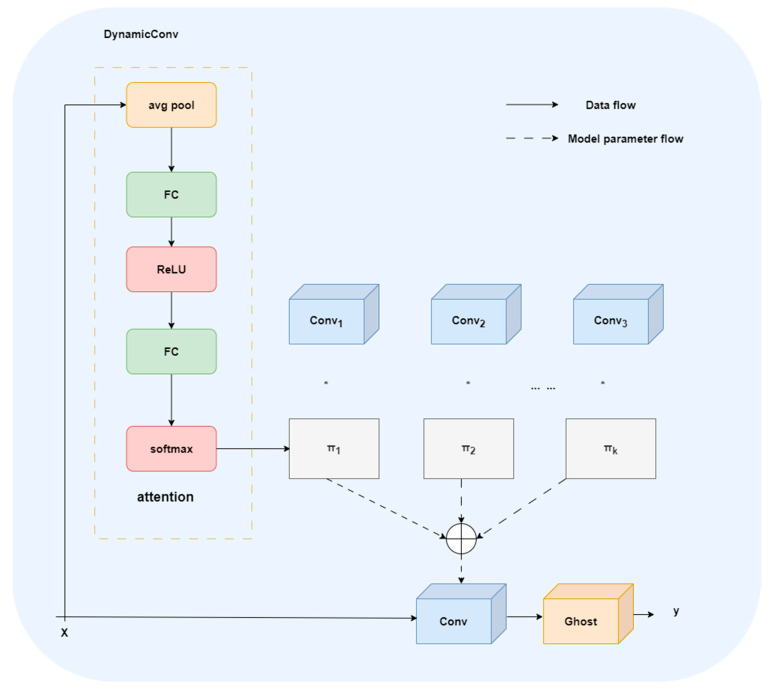
Dynamic convolution.

Dynamic convolution is a dynamic perceptron with adaptive characteristics, which aims to improve the expression ability of the model under the condition of limited computation [20,21,22]. The structural principle is shown in Figure 6. Its structure consists of k shared convolutional kernels of identical size and dimensions. These kernels are dynamically aggregated through attention weights $\left\{\pi_{k\left(x\right)}\right\}$. Subsequently, global average pooling and a fully connected layer generate normalized attention weights. Therefore, dynamic convolution subsequently dynamically selects and aggregates convolution kernels based on the input, thereby enhancing the model’s expressive power. The Ghost module aims to generate an equivalent number of feature maps with fewer parameters, replacing the high computational cost of standard convolutions. Its core idea is to split traditional convolution into two parts: one part uses a small number of conventional convolution kernels to extract basic features; the other part generates additional feature maps from these basic features through low-cost linear transformations. Finally, the output of ordinary convolution is spliced with the features generated by linear transformation to form an output representation equivalent to the original convolution, thus significantly reducing the amount of computation and the number of parameters while maintaining the performance [23].

**Figure 7:**
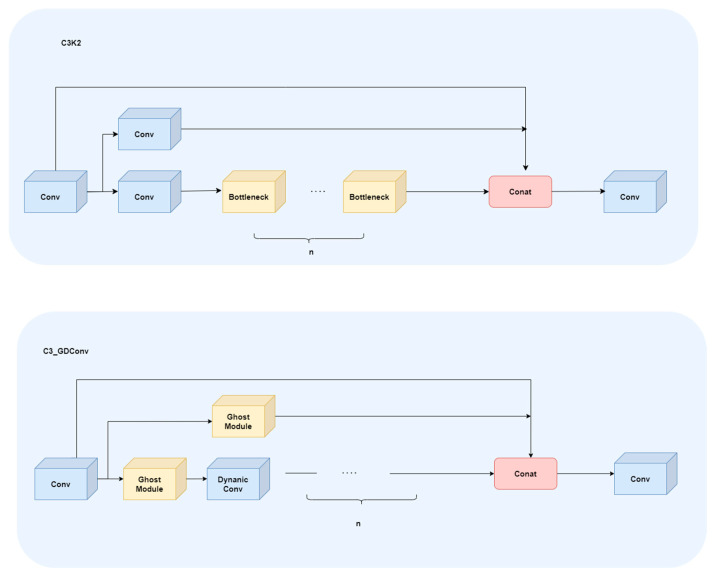
The c3k2 and C3_GDConv modules.

In order to improve the feature modeling ability in small samples and complex scenes, this study introduces the multi-expert mechanism and efficient feature generation strategy of DynamicConv on the basis of C3k2, and constructs C3_GDConv module. Figure 7 shows the structure diagram of C3K2 and C3_GDConv module. C3_GDConv enhances feature representation capabilities through dynamic feature generation and lightweight redundant feature modeling while preserving the original cross-stage partial residual structure (CSP) of C3k2. It is divided into two parallel paths after 1 × 1 convolution dimension reduction. The main branch is stacked by n sub-modules, and each sub-module chooses a different structure according to the parameter c3k setting. When the c3k option is True, the branch internally stacks C3k_GDConv (GhostModule × 2 + DynamicConv):

(5) $$\begin{array}{c} MainBlock=\left\{C3k_{{GDConv}_{1}},\ldots \ldots ,C3{k_{GDConv}}_{n}\right\} \end{array}$$

When the c3k option is set to True, the main branch simplifies to GhostModule stack:

(6) $$\begin{array}{c} MainBlock=\left\{{GhostModule}_{1},\ldots \ldots ,{GhostModule}_{n}\right\} \end{array}$$

The feature generation of GhostModule can be expressed as follows.

(7) $$\begin{array}{c} Y=\left[X\times W\right]⋃\Phi \left(X\right) \end{array}$$

X × W are the ontology features generated by standard convolution. Φ(X) is the redundant (ghost) feature generated by cheap operation (DWConv).

In terms of dynamic convolution design, this paper is inspired by 0mni-Dimensional dynamic convolution (ODConv). In this method, a multi-dimensional attention mechanism (spatial dimension, input channel, output channel, and number of kernels) is used to dynamically generate convolution kernels in a parallel manner [24]. The dynamic generation of convolution kernel weights by ODConv can be formalized as follows.

(8) $$\begin{array}{c} w=\sum_{i=1}^{K}\alpha_{i}\left(x\right)\times w_{i} \end{array}$$

K is the number of expert convolution kernels; w_i the *i*-th expert convolution kernel; α_i (x) is the attention weight based on the input feature x, which satisfies

(9) $$\begin{array}{c} \sum_{i=1}^{K}a_{i}\left(x\right)=1 \end{array}$$

The multi-dimensional attention of ODConv can be expressed as follows.

(10) $$\begin{array}{c} \alpha_{i}=f_{att}\left(x_{spatial},x_{in},x_{out},x_{\ker nel}\right) \end{array}$$

They correspond to spatial dimension, input channel, output channel, and kernel dimension.

In addition, in order to reduce the model parameter overhead caused by dynamic convolution, the pruning + Sparse mechanism of sparse dynamic convolution (SD-Conv) is also borrowed [25]. Sparsity constraints on parallel convolutional branches with learnable masks. The dynamic convolution output is

(11) $$\begin{array}{c} y=\sum_{i=1}^{K}\alpha_{i}\left(x\right)\cdot \left(\tilde{W_{i}}\times x\right) \end{array}$$

In order to solve the problem of feature redundancy, C3_GDConv adopts the fusion mechanism of dynamic convolution and GhostModule. Dynamic convolution dynamically generates convolution kernels through the multi-dimensional attention mechanism, so that each feature selects the most relevant convolution kernel according to its characteristics, avoiding the generation of redundant features. GhostModule generates redundant features through DWConv but only retains the features useful for the task. At the same time, it learns from the sparsity mechanism of SD-Conv and imposes sparse constraints on the parallel convolution branch through learnable masks, which reduces the generation of redundant features, thus effectively controlling the number of redundant features and maintaining the advantages of lightweight network. At the same time, the feature expression ability is improved.

Finally, the main branch features and residual branches were concatenated in the channel dimension, and the output was fused by 1 × 1 convolution. Compared with the C3K2 module, C3k_GDConv significantly improves the expression ability through dynamic convolution while maintaining the lightweight network structure. However, the sparsity mechanism of SD-Conv saves model parameters without significantly increasing the amount of computation. Overall, C3_GDConv has achieved significant improvements in structural lightweight, feature modeling capabilities and task adaptability, providing a more reliable basic feature extraction module for real-time behavior recognition tasks in complex farming environments.

#### 2.2.4. EfficientHead Head

**Figure 8:**
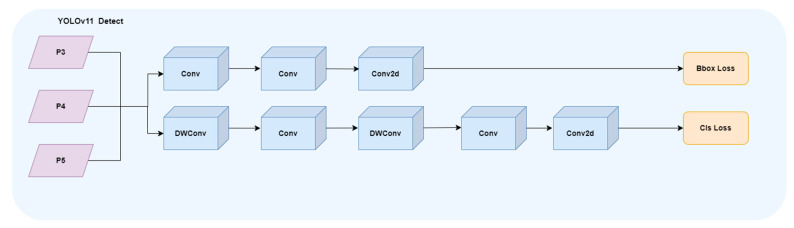
YOLOv11 head detection.

The detection head of YOLOv11 adds two DWConvs to the classification detection head in the original decoupling head of YOLOv8. Figure 8 shows the structure of YOLOv11 detection head, which greatly reduces the amount of parameters and calculation. Although lightweight design improves efficiency, its detection accuracy is not always satisfactory. EfficientHead is a lightweight detection head structure optimized for single-stage object detection tasks, aiming to significantly reduce the computational load while maintaining high accuracy.

It optimizes the detection head of YOLOv8, uses a lightweight 3 × 3 group convolution in the stem module, and integrates multi-channel information through feature splicing, and then uses two 1 × 1 convolution branches to output classification and regression related features. The regression branch uses Distribution Focal Loss (DFL) for bounding box refinement, where the goal is to predict the bounding box offset as a discrete distribution:

(12) $$\begin{array}{c} P=\left\{p_{0,}p_{1K}\right\},\;\sum_{i=0}^{K}p_{i}=1 \end{array}$$

The predicted bounding box is computed by the expectation of distribution as follows.

(13) $$\begin{array}{c} \hat{d}=\sum_{i=0}^{K}i\cdot p_{i} \end{array}$$

Finally, we use dist2bbox to map the distribution prediction to the actual boundary box coordinates for more accurate alignment.

(14) $$\begin{array}{c} \hat{b}=dist2bbox\left(\hat{d}\right) \end{array}$$

**Figure 9:**
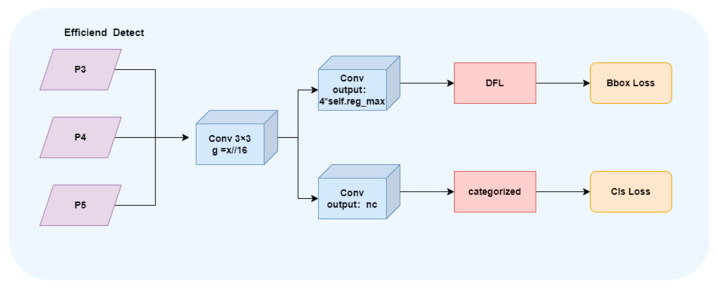
EfficientHead detection head.

In contrast, the Detect structure of YOLOv8 detection head is relatively traditional. The Stem module consists of standard convolutional layers, and then merges features through Concat to output regression and classification prediction. The regression branch does not use DFL, and its speed on bounding box accuracy is weaker than the EfficientHead. The detection head of YOLOv11 extracts features through a lighter Stem, and then directly outputs xywh regression values and class probabilities through independent branches, without DFL operation, which is lighter and faster as a whole, but the accuracy is weaker than the EfficientHead. The EfficientHead provides a good balance between structural complexity and detection accuracy. Compared with YOLOv8, the EfficientHead has higher positioning accuracy and is more efficient, and compared with YOLOv11, it has stronger regression quality. In addition, similar lightweight detection head design has also been proved to effectively improve the positioning accuracy of small targets in remote sensing small target detection tasks [26]. Figure 9 shows the structure diagram of the EfficientHead detection head.

#### 2.2.5. CBAM Attention Mechanism

**Figure 10:**
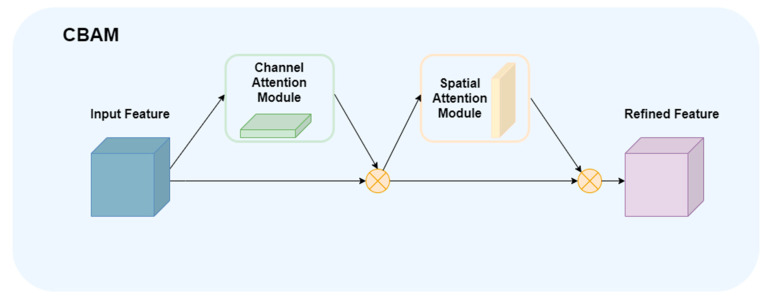
Attention mechanism of CBAM.

In order to further enhance the ability of the model to focus on critical areas in complex environments, CBAM is embedded in the detection head structure. The core idea of CBAM is to separately model “channel importance” and “spatial importance” in CNN, and gradually strengthen the feature map in order [27]. Firstly, the channel weight is obtained by global maximum pooling and average pooling, and the channel weight is obtained by sharing MLP. Then, the channel fusion features are spatially pooled, and the 7 × 7 convolution is performed after the channel splicing. The final output is weighted by the two in turn, which can effectively improve the response ability of the network to the key target area while maintaining very low parameter overhead, and is suitable for embedding in lightweight detection networks and behavior recognition models. It has been proven to effectively improve the performance in a variety of modern detection tasks, such as achieving significant accuracy improvement after applying CBAM in lightweight GoogLeNet [28]. Figure 10 shows the structure diagram of the attention mechanism of CBAM. In this study, in order to further improve the feature expression ability of the model in complex breeding environment, the CBAM attention module is integrated after the C3 module in the 17th layer of the neck network. This layer is in the middle and back segment of the neck network, and the generated feature map not only carries rich semantic information of deep features, but also retains fine spatial details of shallow features, which makes it an ideal location for the attention mechanism. In theory, the attention module is more suitable to be placed at the feature layer that can take into account both semantic expression and spatial resolution, so as to effectively enhance the response of key behavior regions. By applying channel and spatial attention in parallel, CBAM can adaptively calibrate channel weights and focus on key spatial regions, thereby enhancing the ability of the model to distinguish behavioral features. It is placed after the C3 module of the 17th layer, which can make full use of the semantic depth and spatial integrity of the features of this layer, and realize the accurate capture of behavioral features in complex environments.

## 3. Results

### 3.1. Experimental Platform and Parameter Settings

In this study, the image input size is set to 640 × 640. Stochastic gradient descent algorithm (SGD) was used for training, the training cycle was 250 rounds, the training batch size was set to 8, the workers were set to 4, and Mosaic data augmentation was enabled throughout. All the experiments are implemented on a Windows system, and the specific experimental environment configuration is shown in Table 3.

### 3.2. Evaluation Metrics

In this study, precision, recall, number of floating-point operations per second, and number of parameters were used to evaluate the performance of sika deer behavior recognition in a farmed environment.

Precision is the proportion of positive samples predicted by the model that were actually positive. Where TP, FP, FN represent the number of true, false positive, and false negative examples, respectively.

(15) $$\begin{array}{c} \mathrm{Precision}=\frac{TP}{TP+FP} \end{array}$$

Recall is the proportion of samples that were actually positive that were correctly predicted as positive by the model.

(16) $$\begin{array}{c} \mathrm{Recall}=\frac{TP}{\left(TP+FN\right)} \end{array}$$

mAP is the average value of AP across multiple categories, used to measure the detection performance of a model on the entire dataset.

(17) $$\begin{array}{c} mAP=\frac{1}{N}\sum_{i=1}^{N}AP_{i} \end{array}$$

FLOPs refers to the amount of computation, K is the size of the convolution kernel, C_in is the number of input channels, C_(out) is the number of output channels, H and W denote the height and width of the output feature map, respectively.

(18) $$\begin{array}{c} FLOPs=\sum \left(K\times K\times C_{in}\times C_{out}\times H\times W\right) \end{array}$$

Params refers to the total number of parameters that need to be trained in a model.

(19) $$\begin{array}{c} Params=\sum \left(K\times K\times C_{in}\times C_{out}\right) \end{array}$$

#### 3.2.1. Comparison of Different Detection Models

**Figure 11:**
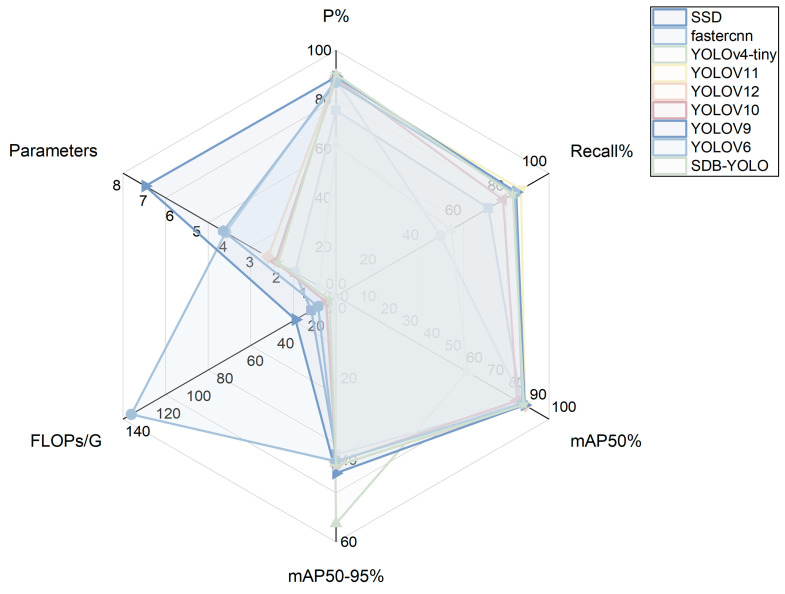
Radar diagram of target detection results of different algorithms.

In order to comprehensively evaluate the performance of the proposed method, this paper selects six representative object detection models as comparison objects from the perspectives of computational efficiency, model lightweight degree and practical application feasibility. These include SSD, Faster R-CNN, YOLOv4-Tiny as well as YOLOv6, YOLOv9, and YOLOv11. Among them, SSD and Faster R-CNN represent the typical structures of single-stage and two-stage object detection methods, respectively. YOLOv4-Tiny is a lightweight YOLO series model, which is widely used in resource-constrained scenes. YOLOv6, YOLOv9, and YOLOv11 represent different development directions of YOLO series in structural design and performance optimization in recent years. The above models are representative in terms of computational complexity, detection accuracy, and deployment characteristics, which help to verify the comprehensive performance advantages of the proposed method in small-sample and smart aquaculture application scenarios from multiple perspectives. All the comparison models were trained from scratch, using the default hyperparameter configuration provided by the official, and completed training and testing under the same dataset division and unified hardware environment to ensure the fairness of the experimental process and the comparability of results. The results are shown in Table 4, while the radar chart shown in Figure 11 visually shows the comprehensive performance of each model on the six dimensions. Among them, the four dimensions of precision (P%), Recall (Recall%), mAP50, and MAP50-95 have better effects as the polyline is closer to the outside, while FLOPs and the number of parameters have better lightweight effects as they are closer to the inside of the circle.

After comprehensive evaluation of each model, YOLOV11 was selected as the most appropriate benchmark model in this study. YOLOV11 has a balanced performance in precision (87.1%), recall (86.9%), and mAP50 (88.7%). At the same time, YOLOv11 has a lower computational overhead (6.3 GFLOPs) and a smaller parameter amount (about 2.58 M), which is more efficient than other models while maintaining high accuracy. Therefore, YOLOV11 achieves the best balance between accuracy and resource consumption, and is suitable as a benchmark for subsequent optimization and application.

Based on this, the SDB-YOLO model proposed in this study has achieved further improvements compared to the original YOLOV11: the number of floating-point numbers calculated has been reduced by 2.0 G, the number of parameters has been reduced by 4.09 × 10^5^, while the accuracy has been improved by 3.1% and the mAP50-95 has been enhanced by 0.2%. Combined with the radar chart shown in Figure 9, it can be seen more intuitively that SDB-YOLO significantly reduces the model complexity while maintaining high accuracy, and achieves a better balance between lightweight and performance. Therefore, SDB-YOLO shows the advantages of high accuracy and lightweight, and is the best model choice after improvement.

Table 5 compares the performance differences between SDB-YOLO proposed in this study and YOLOv11 in terms of accuracy (P%), detection speed (FPS), and model size (MB). The results show that SDB-YOLO achieves improvement in accuracy, indicating that the lightweight design can enhance the detection performance while retaining the expression ability of key features. More significantly, the model size of SDB-YOLO is only 4.58 MB, which is greatly reduced compared with 15.84 MB of YOLOv11, which fully reflects the advantages of portability and is helpful for deployment in storage limited or edge computing environments. Although the detection speed is slightly lower than YOLOv11, in practical applications, the storage and energy consumption advantages brought by the lightweight model can effectively compensate for the speed reduction and provide a more flexible deployment scheme in complex breeding environments. Overall, the experimental results quantitatively verify the balance between portability and detection performance of SDB-YOLO, which provides a reliable basis for the application of the model in resource-constrained scenarios.

**Figure 12:**
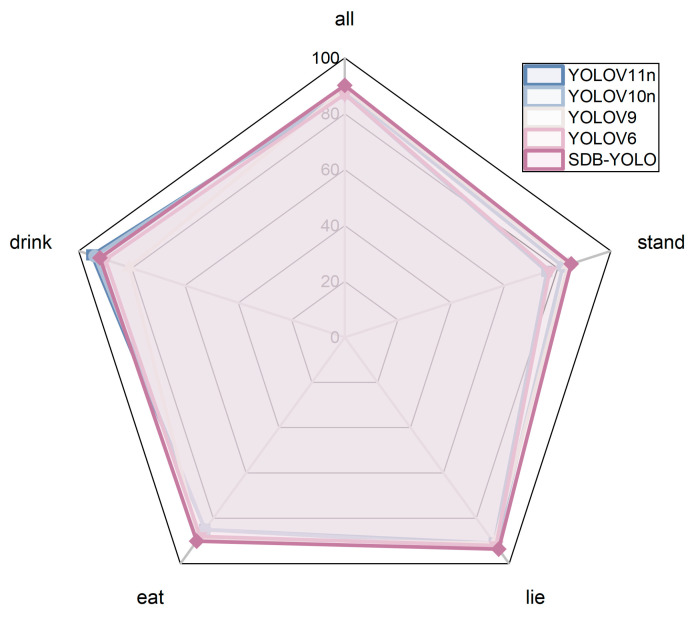
Accuracy metrics for the four behaviors and overall for the five models.

For a more comprehensive comparison, Table 6 shows the accuracy of the five models and the improved model on the four behaviors. The results show that the accuracy of the improved model is greatly improved in the overall and three behaviors. We also drew a radar chart for visual comparison, and the results are shown in Figure 12. This figure comprehensively evaluates YOLOV6, YOLOV9, YOLOV10n, YOLOV11n, and the SDB-YOLO model proposed in this study from five dimensions (all, stand, sit, eat, drink). The area and contour of each polygon intuitively reflect the comprehensive performance and balance of the model in each category. In general, all the models in the comparison show high and similar performance levels on the five dimensions, indicating that SDB-YOLO can handle these common behavior categories well. It is worth noting that the polygonal curves representing the SDB-YOLO model are closest to the periphery in multiple categories (especially “drink”, “lie”, and “eat”), indicating that it has achieved optimal or near-optimal performance in most scenarios, demonstrating its outstanding detection ability and good category balance.

In conclusion, the results show that the SDB-YOLO model proposed in this study maintains a comprehensive performance (all) comparable to that of the cutting-edge YOLO series models while having competitive advantages in some subcategories, verifying the effectiveness of the model improvement.

#### 3.2.2. Comparison of Improved Module Performance

**Figure 13:**
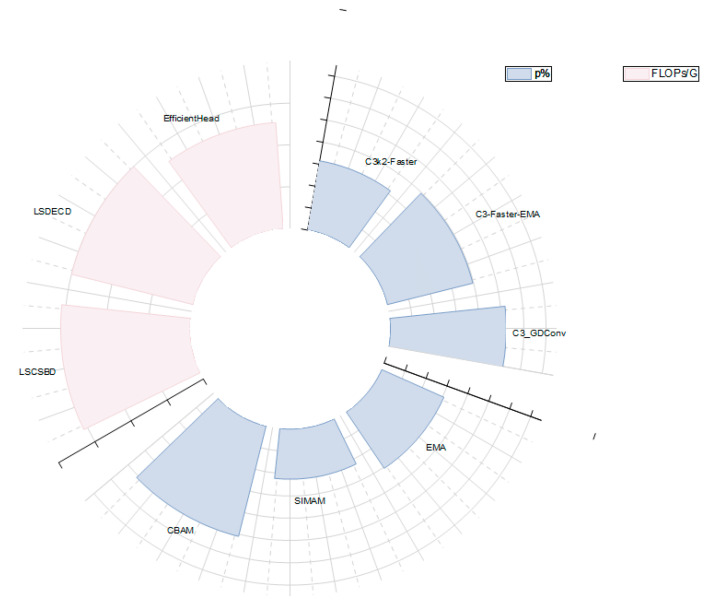
Comparison radar chart of performance indicators of different modules.

In this study, in order to further explore the effectiveness of C3_GDConv to improve C3K2 module, three groups of comparative experiments are designed under the same experimental conditions, using c3K2-Faster, C3-Fare-EMA, and C3_GDConv proposed in this paper [29,30,31]. The corresponding experimental results are listed in the first three rows of Table 7. It can be seen from the results that C3_GDConv achieves the highest detection accuracy (90.4%) among the three improved modules, and its computational complexity is the lowest, only 5.4 GFLOPs, indicating that the module has better comprehensive performance in accuracy and lightweight. Figure 13 intuitively shows the comparison results of this group in the form of radial bar chart, and it can be seen that C3_GDConv is better than the other two modules in terms of accuracy related indicators as a whole.

In order to further evaluate the influence of different detection head structures on the performance of the model, three lightweight detection heads, LSCSBD, LSDECD, and EfficientHead, are selected for comparison experiments, and the results are shown in Table 7. Combined with the corresponding radial bar comparison in Figure 11, it can be found that under the premise of ensuring the detection accuracy, the EfficientHead performs better in terms of computational complexity and model scale, and its lightweight effect is better than LSCSBD and LSDECD.

In addition, in order to improve the expression ability of the model in the feature fusion stage, EMA, SimAM, and CBAM attention mechanisms are introduced into the neck structure for comparative experiments. The correlation results are also presented uniformly in the radial bar plots of FIG. 11. The experimental results show that the performance of the model is improved after introducing the attention mechanism, among which CBAM is the most prominent in a number of evaluation indicators, and the detection accuracy is improved to 90.2%, which is better than EMA and SimAM.

### 3.3. Ablation Experiments

In order to further explore the effectiveness of YOLOV11 network improvement in this study, nine groups of ablation experiments are designed. The improvement of SDB-YOLO model mainly includes FPSC, C3_GDConv in the backbone, C3_GDConv in the neck, CBAM, and EfficientHead detection head. Consistent hardware environment and model training parameter settings were maintained in ablation experiments. The relevant results of different algorithm combinations are shown in Table 8. The results show that compared with YOLOV11, the improved SDB-YOLO algorithm can improve the accuracy on the basis of lightweight. The results clearly show that the original YOLOV11 model still has room for improvement in the behavior recognition of small samples of sika deer in the breeding environment, and it is significantly effective in four method improvements:

(1) After introducing the EfficientHead, the detection performance of the model remains stable as a whole, while the computational complexity is significantly reduced. Table 8 shows that GFLOPs decreases from 6.3 to 5.1, and the number of parameters also decreases synchronously, indicating that the EfficientHead effectively reduces the computational overhead of the model without significantly affecting the detection accuracy.
(2) After introducing C3_GDConv into the backbone network and neck structure, the feature extraction and representation ability of the model is enhanced, and the overall detection accuracy is improved by 3.3%, which verifies the effectiveness of the module in feature expression enhancement.
(3) The FPSC module highlights its advantages in feature fusion and computational efficiency. It can be seen that after adding the FPSC module, the accuracy is increased by 3.4%, the MAP50 is increased by 0.9%, and the MAP50-95 is increased by 0.2%, which further proves that it can play an important role in multi-scale feature fusion.
(4) SDB-YOLO achieves high accuracy while maintaining low computational cost, and the accuracy is further improved after adding the CBAM attention mechanism. The multi-module collaboration and attention mechanism effectively optimize the overall model performance, which verifies the rationality and effectiveness of SDB-YOLO.

**Figure 14:**
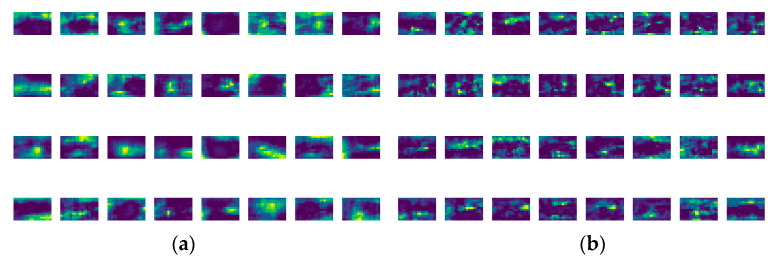
FPSC module characteristics of multi-scale map visualization compared before and after improvement. (**a**) shows the feature map output by the YOLOv11 model, and the right (**b**) shows the corresponding output of the improved SDB-YOLO.

Figure 14 shows the comparison of multi-scale feature maps on sika deer pose recognition task before and after the improvement of FPSC module. The left (a) shows the feature map output by the YOLOv11 model, and the right (b) shows the corresponding output of the improved SDB-YOLO. Both sets of feature maps are visualized as 32 channels (4 rows ×8 columns), with dark colors indicating low responses and light to yellow–green areas indicating high activation responses. By comparison, it can be seen that after replacing the SPPF module with FPSC, the spatial distribution of feature responses is more concentrated and coherent, and the interference responses of background and irrelevant textures are significantly reduced. The improved FPSC module can make the model have a feature representation with higher discrimination and clearer structure, which is helpful to improve the accuracy of subsequent analysis.

**Figure 15:**
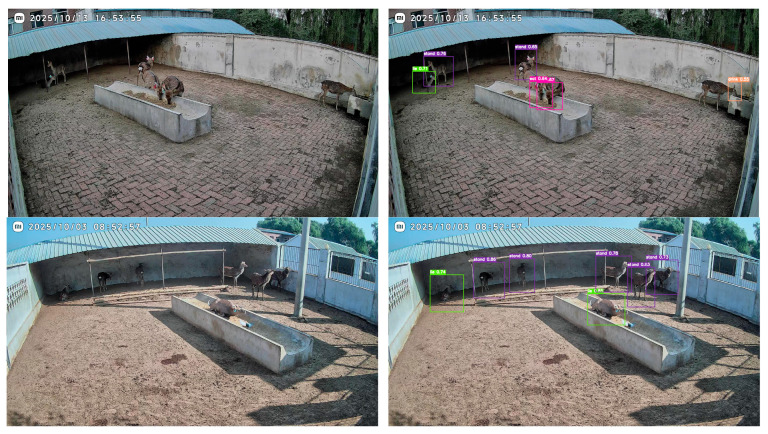
SDB-YOLO model detection results.

Figure 15 shows the detection results of the improved SDB-YOLO model in a real farming scenario. It can be observed from the figure that the model can give the detection box and the corresponding behavior category label for multiple sika deer at the same time in different shooting angles and activity areas. For the behaviors such as standing, eating, and lying on the stomach, the corresponding confidence values of the detection results are mainly distributed in the range of 0.55 to 0.9. When the individuals are partially occluded, close to each other or located at the far end of the screen, the detection and pose identification of different sika deer individuals can still be given, respectively, and there is no obvious detection loss or category confusion.

### 3.4. Thermal Analysis Diagram

**Figure 16:**
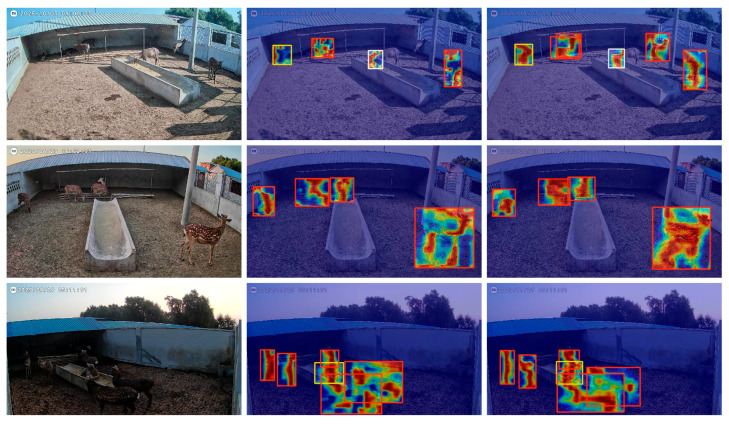
Comparison of heat maps before and after model optimization.

Figure 16 shows the heat map comparison between the original YOLOv11 model and the improved SDB-YOLO model in the sika deer behavior detection task. It can be seen that the YOLOv11 model has a weak response in some local areas, which leads to an inaccurate localization of sika deer targets. In the first row of images, a standing sika deer on the right is missed in YOLOv11, while SDB-YOLO successfully detects it. In the comparison of the second and third rows, SDB-YOLO can still effectively detect sika deer in the case of dense distribution, and the distribution of high-response areas in Figure X(c) is more concentrated and the boundaries are more coherent. This shows that the improved SDB-YOLO model has better performance in capturing multi-scale detail features and fusing spatial information.

## 4. Discussion

In this study, a lightweight SDB-YOLO behavior recognition model was constructed for small-sample breeding environment, which was used to recognize four basic behaviors of sika deer: standing, lying down, eating, and drinking. Experimental results show that the proposed model can obtain high recognition accuracy on the self-built dataset, while maintaining low parameter scale and computational complexity. This shows that under the condition of small sample size, the visual features related to deer behavior can still be extracted more stably through reasonable network structure design. The relevant results provide a reference for the research of animal behavior recognition under resource-constrained conditions, and also provide a feasible idea for behavior monitoring in small-sample breeding scenarios.

According to the actual needs of breeding management, the model in this paper has a certain application potential in animal health monitoring. Animal behavior changes are usually important external manifestations of health status and stress level. Through continuous monitoring of basic behaviors such as standing, lying down, eating, and drinking, individual behavior patterns can be quantified and analyzed. For example, a decrease in feeding or drinking behavior, an abnormally prolonged time spent lying down, and a decrease in overall activity levels are often associated with decreased body condition, illness, or stress reactions. The behavior information obtained based on the proposed method can provide more intuitive reference for farmers, help to find potential abnormal individuals, and thus provide a basis for subsequent manual intervention and management decisions.

A variety of influencing factors still need to be considered when applying this method in a real farming environment. The large variation in light conditions, frequent occlusion between individuals and obvious differences in camera perspective may affect the recognition results of the model. At present, the models are mainly trained on limited scene data, and the recognition performance may fluctuate when directly used in different farming areas or under different environmental conditions. However, due to its lightweight structure and low computation and storage overhead, the model still has the feasibility of long-term operation on edge devices. The stability of the model in practical applications is expected to be further improved by introducing more data from aquaculture scenarios and making targeted adjustments combined with the specific environment.

Although the proposed method shows a balance between recognition accuracy and model size, there is still room for further optimization. The current behavior category mainly covers basic actions, and does not include highly dynamic or abnormal behavior types such as running and attacking, so it needs to be extended to fully reflect the behavioral characteristics of deer. The data sources are relatively concentrated, and the generalization ability of the model in different breeding scenarios still needs to be further verified. At the same time, the lightweight design significantly reduces the size of the model, but the detection speed (FPS) is slightly reduced compared with some large models, which may have a certain impact on the system response efficiency in high-frame-rate real-time monitoring scenarios. Future research can introduce abnormal behavior analysis and time series modeling methods on the basis of expanding behavior categories, and further optimize the inference efficiency of the model, so as to enhance its application potential in animal health monitoring and smart farming management.

## 5. Conclusions

In view of the limited number of samples, significant differences in individual behavior, and high demand for continuous monitoring in the breeding environment, a lightweight behavior recognition method for sika deer is proposed in this paper. This method realizes non-contact behavior recognition based on images, which can effectively represent key behavior characteristics under small-sample conditions, reduce the influence of insufficient samples and individual differences on recognition performance, and improve the stability and availability of behavior recognition in complex breeding environments. The results show that the constructed recognition model can provide reliable behavioral data support for sika deer health status monitoring, feeding and breeding management, and animal welfare assessment. The analysis of feeding and activity levels can assist in identifying potential health risks and stress changes, thus providing technical support for fine breeding management and animal welfare improvement.

Based on the YOLOv11 architecture, we innovatively designed the SDB-YOLO model. This model features a novel FPSC module that effectively balances feature fusion and computational efficiency, significantly enhancing the network’s overall feature modeling capability. Subsequently, we constructed the C3_GDConv module to further enhance feature extraction capabilities, enabling the model to achieve sufficient and robust expressive power even under small-sample conditions. In addition, the CBAM attention module is introduced in the neck, which effectively strengthens the model’s ability to focus on key behavior areas and improves the identification accuracy of fine-grained behavior differences. Finally, the detection head was replaced with the lightweight EfficientHead, which reduces redundant computation and enables the model to maintain high efficiency while remaining lightweight. Experimental results demonstrate that the SDB-YOLO model achieves outstanding detection accuracy across all four typical behavioral recognition tasks for sika deer. It exhibits a balanced advantage of both lightweight architecture and high precision in comprehensive performance evaluation, holding significant practical implications for the advancement of smart farming.

## Figures and Tables

**Table 1:** The most advanced work in the field.

Study	Behavior	Methods	Result
Duck	Drinking, lying, standing, preening, spreading, and eating	YOLOv5s	mAP@0.5:0.95 increased by 2.3%
Lamb	Running, sleep, socialization, wandering, excitement, hungry, lethargy, and normal	YOLOv8n	While maintaining a higher mAP, the parameters were reduced by 36.6% and the GFLOPs by 30% 0.5
Piglet	Lying, kneeling, standing, drinking, suckling, trampling, hitting, and biting ear	YOLOv8	The precision is increased by 2.5%, the Mean Average Precision (mAP) is increased by 1.5%, the inference delay is reduced by 9.1 ms
Chicken	Standing, lying, eating, drinking, exploring, gasping, and grooming	YOLO11s	MAP increased by 2.2%
Cow	Eating, drinking water, lying down, and standing	YOLOV3	The accuracy rate of 90.4% surpasses the detection results of models such as YOLOv3 and YOLO-tiny by 1.2% and 12.9%, respectively.

**Table 2:** Detailed descriptions about different behaviors.

Behavior	Description of Behavior	Number of Labels
stand	The limbs are straight to support the body, the torso is off the ground, the head can be neutral, bowed, or raised, and the individual is in a static or alert state.	2369
lie	Front leg kneeling, chest, abdomen, buttocks on the ground, lying posture for the body arched half side lying.	812
eat	Bend your head in front of the cement tank or forage area, and use your lips and tongue to take in the feed precisely.	1600
drink	Bend your head and stretch your neck next to an artificial sink, touching and ingesting water through your mouth and nose.	281

**Table 3:** Experimental hardware environment.

Environmental Configuration	Parameters
Operating system	Windows
CPU	Intel(R)Core(TM)i9-10920X CPU@3.50 GHz
GPU	NVIDIA GeForce RTX 3080
Framework	PyTorch 2.0.0
Language	Python 3.8.20
Development environment	PyCharm 2023.2.5

**Table 4:** Comparison of object detection results across different algorithms.

Models	P%	Recall%	mAP50%	mAP50-95%	FLOPs/G	Parameters
SSD	75.5	71.5	86.5	39.3	16.3	1,543,000
fastercnn	88	49.1	88.3	40.29	134.6	4,145,000
YOLOv4-tiny	61.6	54.1	61.6	55.5	1.9	632,000
YOLOV11	87.1	86.9	88.7	41.3	6.3	2,582,932
YOLOV12	89.6	83.3	88.9	41.4	6.3	2,557,508
YOLOV10	88.5	78.4	85	40.5	6.5	2,265,984
YOLOV9	89.2	84.6	88.5	43.2	26.7	7,168,636
YOLOV6	87	84	87.5	40.3	11.8	4,234,140
SDB-YOLO	90.2	83	88.2	41.5	4.3	2,174,390

**Table 5:** Comparison of the indicators of the model before and after improvement.

Models	P%	Detection Speed (FPS)	Model Size (MB)
YOLOV11	87.1	77.6	15.84
SDB-YOLO	90.2	47.8	4.58

**Table 6:** Accuracy of six representative models under different behaviors of sika deer.

Models	All	Stand	Lie	Eat	Drink
11n	87.1	76.4	91.5	85	95.3
10n	88.5	82.3	92.5	85.2	94.1
9	89.2	84.5	91.7	89	81.9
6	87	77.2	92.1	88.1	90.6
SDB-YOLO	90.2	85.1	93.6	90.1	92

**Table 7:** Performance metrics of different modules.

	P%	Recall%	mAP50%	mAP50-95%	FLOPs/G
C3k2-Faster	86.3	81.6	87.8	40.5	5.8
C3-Faster-EMA	87.9	81.6	87.5	40.9	5.9
C3_GDConv	90.4	80.5	88.7	42.2	5.4
LSCSBD	87.8	85.2	87.9	41.8	6.2
LSDECD	86.2	83	88.1	40.9	6.0
EfficientHead	87.5	83.9	87.5	41.2	5.1
ema	86.1	85	87.5	41.2	4.4
simam	84.5	80.8	86.5	40.6	4.3
cbam	90.2	83	88.2	41.7	6.5

**Table 8:** Ablation experiment results of different optimization modules.

EfficientHead	FPSC	C3_GDConv	CBAM	P	R	MAP50	MAP50-95	GFLOPS	Parameters
				87.1	86.9	88.7	41.3	6.3	2,582,932
√				87.5	83.9	87.5	41.2	5.1	2,313,492
		√		90.4	80.5	88.7	42.2	5.4	2,227,412
	√			90.5	84.9	89.6	41.5	6.3	2,730,388
√	√			86.7	82.5	87.2	41	5.1	2,460,947
	√	√		88.9	81.2	87.6	41	5.5	2,376,260
√		√		87.6	80.5	86.9	41.1	4.3	1,959,364
√	√	√		89.6	82.3	88.7	41.2	4.3	2,106,820
√	√	√	√	**90.2**	83	88.2	**41.5**	**4.3**	**2,174,390**

## Data Availability

The behavioral video and image data of sika deer in this study are not stored in public databases due to commercial confidentiality of the collaborating breeding farms and confidentiality agreements. Relevant data can be obtained from the corresponding author upon reasonable request.
